# From Weight Loss to Organ-Specific Disease Modification: A Narrative Review of Incretin-Based Therapies in Obesity-Related Cardiometabolic Disease, Chronic Kidney Disease, Metabolic Dysfunction-Associated Steatohepatitis, and Obstructive Sleep Apnoea

**DOI:** 10.7759/cureus.112135

**Published:** 2026-07-06

**Authors:** Devarsh Joshi

**Affiliations:** 1 General Medicine, Basingstoke and North Hampshire Hospital, Basingstoke, GBR

**Keywords:** cardiovascular disease, chronic kidney disease (ckd), glp-1 receptor agonists, heart failure with preserved ejection fraction, mash, obesity, obstructive sleep apnoea, semaglutide, tirzepatide

## Abstract

Incretin-based therapies have changed the management of type 2 diabetes and obesity. Semaglutide acts through the glucagon-like peptide-1 receptor, while tirzepatide combines glucose-dependent insulinotropic polypeptide and glucagon-like peptide-1 receptor agonism. These therapies were initially used mainly for glycaemic control and weight reduction, but recent evidence suggests that their role now extends into obesity-related organ disease, including atherosclerotic cardiovascular disease, heart failure with preserved ejection fraction, chronic kidney disease, metabolic dysfunction-associated steatohepatitis, and obstructive sleep apnoea.

This review summarizes evidence for semaglutide and tirzepatide across obesity-related cardiometabolic, renal, and hepatic outcomes, as well as obstructive sleep apnoea-related outcomes. A targeted literature search was conducted using PubMed, MEDLINE, major journal websites, and regulatory sources from database inception to May 16, 2026. Current evidence supports clinically important benefits in selected patients, although the strength of evidence varies by condition. Important limitations include treatment durability, weight regain after discontinuation, adverse effects, peri-procedural considerations, pregnancy avoidance, cost, drug supply, and unequal access. These therapies should therefore be viewed as potential components of chronic disease management for selected patients with obesity, type 2 diabetes, or obesity-related cardiometabolic, renal, hepatic, or sleep-disordered breathing complications, rather than as short-term weight-loss interventions.

## Introduction and background

Obesity is increasingly recognized as a chronic disease with effects that extend well beyond body weight alone. Excess or dysfunctional adiposity can contribute to impairment at tissue, organ, and whole-person levels [1]. Clinically, this matters because many patients with obesity do not present with obesity in isolation. They often have overlapping metabolic, cardiovascular, renal, hepatic, respiratory, and functional complications, including type 2 diabetes, atherosclerotic cardiovascular disease, heart failure, chronic kidney disease, metabolic dysfunction-associated steatotic liver disease (MASLD), obstructive sleep apnoea, impaired quality of life, and increased mortality risk. Although lifestyle intervention remains central to care, lifestyle advice alone is often insufficient for patients who already have established obesity-related complications.

Incretin-based therapies have changed the treatment landscape. Glucagon-like peptide-1 receptor agonists (GLP-1 RAs) were first developed for type 2 diabetes, with effects on glucose-dependent insulin secretion, glucagon suppression, gastric emptying, appetite, and food intake [2,3]. More recently, these therapies have become central to pharmacological obesity treatment. Tirzepatide, a dual glucose-dependent insulinotropic polypeptide (GIP) and GLP-1 receptor agonist, has added to this field by producing substantial effects on both glycaemic control and body weight [4].

The clinical evidence has developed quickly. Semaglutide 2.4 mg and tirzepatide 5 mg, 10 mg, and 15 mg once weekly have produced substantial weight reduction in large obesity trials [5,6]. Withdrawal and continuation studies have also shown that ongoing treatment is usually needed to maintain benefit [7,8]. At the same time, the role of incretin-based therapy has expanded beyond weight reduction. Cardiovascular outcome trials first established the value of GLP-1 RAs in type 2 diabetes [9-11], and the SELECT trial later showed cardiovascular benefit with semaglutide in patients with established cardiovascular disease and overweight or obesity, but without diabetes [12]. Further evidence and regulatory developments have supported the relevance of incretin-based therapy in obesity-related heart failure with preserved ejection fraction (HFpEF), chronic kidney disease, metabolic dysfunction-associated steatohepatitis (MASH), and obstructive sleep apnoea [13-23].

This review examines that shift: from incretin-based therapies as glucose-lowering or weight-loss drugs to treatments that may influence obesity-related organ disease. The central question is not only whether these therapies reduce body weight but also whether they improve clinically meaningful outcomes in obesity-related disease states. This includes cardiovascular event reduction, heart failure symptoms and physical function, kidney outcomes, liver histology, and obstructive sleep apnoea severity. The aim is not to suggest that these agents replace established disease-specific care, but to consider how recent evidence changes their clinical positioning. This is particularly relevant for patients with obesity-related multimorbidity, where cardiovascular, renal, hepatic, and sleep-related disease often overlap. The review therefore brings together recent evidence across these disease areas, while also highlighting where benefits may be mediated by weight loss, where additional incretin-related mechanisms may contribute, and where uncertainty remains. The key studies and regulatory sources supporting this expansion of incretin-based therapy are summarized in Table 1.

**Table 1:** Landmark evidence supporting the expansion of incretin-based therapy beyond weight loss. HFpEF: heart failure with preserved ejection fraction; MASH: metabolic dysfunction-associated steatohepatitis.

Clinical area	Study or source	Therapy	Population	Main relevance
Obesity	STEP 1 [5]	Semaglutide 2.4 mg	Adults with overweight or obesity without diabetes	Demonstrated substantial weight reduction compared with placebo
Obesity	SURMOUNT-1 [6]	Tirzepatide	Adults with overweight or obesity without diabetes	Demonstrated large dose-dependent weight reduction
Weight maintenance	SURMOUNT-4 [8]	Tirzepatide	Adults with obesity after tirzepatide lead-in	Continued therapy maintained weight loss; withdrawal led to regain
Cardiovascular disease	SELECT [12]	Semaglutide 2.4 mg	Established cardiovascular disease with overweight or obesity without diabetes	Reduced major adverse cardiovascular events
HFpEF	STEP-HFpEF [13]	Semaglutide 2.4 mg	HFpEF with obesity	Improved symptoms, physical limitations, exercise function, and weight
HFpEF with diabetes	STEP-HFpEF DM [14]	Semaglutide 2.4 mg	HFpEF with obesity and type 2 diabetes	Improved symptoms, physical limitations, and weight
HFpEF	SUMMIT [15]	Tirzepatide	HFpEF with obesity	Reduced cardiovascular death or worsening heart failure composite
Chronic kidney disease	FLOW [16]	Semaglutide 1.0 mg	Type 2 diabetes with chronic kidney disease	Reduced clinically important kidney outcomes and cardiovascular death
MASH	SYNERGY-NASH [18]	Tirzepatide	MASH with moderate or severe fibrosis	Improved MASH resolution without worsening fibrosis
MASH	ESSENCE [19]	Semaglutide 2.4 mg	MASH with fibrosis	Improved steatohepatitis resolution and fibrosis outcomes
Obstructive sleep apnoea	SURMOUNT-OSA [22]	Tirzepatide	Moderate-to-severe obstructive sleep apnoea with obesity	Reduced apnoea-hypopnoea index and improved related outcomes

## Review

Literature search strategy

This review was prepared using a targeted search of PubMed, MEDLINE, major journal websites, and regulatory sources from database inception to May 16, 2026, with no lower date restriction applied. Search terms included “GLP-1 receptor agonist,” “semaglutide,” “tirzepatide,” “GIP GLP-1 receptor agonist,” “obesity,” “cardiovascular outcomes,” “heart failure with preserved ejection fraction,” “chronic kidney disease,” “metabolic dysfunction-associated steatohepatitis,” “MASH,” “NASH,” “obstructive sleep apnoea,” “weight regain,” “gallbladder disease,” “safety,” “clinical obesity,” “MASLD guideline,” “weight management obstructive sleep apnoea,” “pregnancy semaglutide tirzepatide,” and “perioperative GLP-1 receptor agonist guidance.”

Priority was given to randomized controlled trials, cardiovascular outcome trials, phase 2 and phase 3 clinical studies, systematic reviews, clinical practice guidelines, regulatory updates, and prescribing information where relevant. Studies and sources were included if they reported clinical efficacy, safety, mechanistic, regulatory, or guideline information relevant to incretin-based therapies in obesity-related disease. Preclinical studies, duplicate reports, conference abstracts without full peer-reviewed publication, and articles not directly relevant to incretin-based therapy or obesity-related disease were excluded unless they provided essential background context. Evidence was synthesized descriptively by disease area rather than statistically. This article is a narrative review rather than a systematic review. A formal Preferred Reporting Items for Systematic Reviews and Meta-Analyses flow diagram, risk-of-bias assessment, and meta-analysis were not performed. The purpose was to provide a clinically focused synthesis of high-impact evidence and to discuss how incretin-based therapies are being repositioned across obesity-related multimorbidity.

Mechanistic basis for benefits beyond weight loss

The benefits of incretin-based therapy are unlikely to be explained by reduced calorie intake alone. GLP-1 RAs act through pancreatic, gastrointestinal, neural, and possibly cardiovascular and renal pathways. Their pancreatic effects include increased glucose-dependent insulin secretion and reduced glucagon secretion. Their gastrointestinal and central effects reduce appetite, meal size, food reward, and energy intake [2,3].

A key issue is whether the benefits of incretin-based therapies are explained entirely by weight loss. Current evidence suggests a mixed picture. Some benefits are likely to be strongly weight-mediated, particularly improvements in obesity-related HFpEF, MASH, and obstructive sleep apnoea. However, other findings suggest broader cardiometabolic and organ-specific effects. For example, cardiovascular benefit has been shown in patients with established cardiovascular disease and overweight or obesity without diabetes, and kidney outcome data suggest effects on albuminuria and cardiorenal risk that may not be explained by weight reduction alone [11,12,16]. Therefore, the most clinically useful interpretation is that incretin-based therapies act through both weight-dependent and potentially weight-independent pathways, with the relative contribution varying by disease state.

Weight reduction itself can improve several disease pathways. Loss of visceral adiposity can improve insulin resistance, blood pressure, systemic inflammation, hepatic fat accumulation, ventricular loading conditions, and upper-airway mechanics. These changes are highly relevant to type 2 diabetes, HFpEF, chronic kidney disease, MASH, and obstructive sleep apnoea. However, it would be too simplistic to assume that all clinical benefits are mediated only by weight loss. GLP-1 RAs may also influence inflammation, endothelial function, natriuresis, albuminuria, and atherosclerotic plaque biology [2,11].

Tirzepatide combines GIP and GLP-1 receptor agonism. The additional contribution of GIP receptor activation is still being clarified, but dual agonism is associated with large reductions in weight and improvements in glycemic parameters [4,6]. Whether the organ-specific benefits of tirzepatide are fully explained by weight reduction or partly reflect direct incretin effects remains uncertain. This matters clinically because it may affect how these drugs are selected for patients with different patterns of obesity-related disease.

Weight reduction and maintenance

The STEP 1 trial was a landmark study for pharmacological obesity treatment. Adults with overweight or obesity without diabetes were randomized to once-weekly semaglutide 2.4 mg or placebo alongside lifestyle intervention. At 68 weeks, semaglutide produced a mean body-weight reduction of 14.9%, compared with 2.4% with placebo [5]. This magnitude of weight loss was far greater than that observed with many older anti-obesity medications and provided strong evidence that pharmacotherapy could achieve clinically significant weight reduction across a broad population with obesity.

Tirzepatide produced even larger weight reductions in SURMOUNT-1. In adults with obesity or overweight without diabetes, the mean percentage weight change at 72 weeks was 15.0%, 19.5%, and 20.9% with tirzepatide 5 mg, 10 mg, and 15 mg, respectively, compared with 3.1% with placebo [6]. This placed tirzepatide at the upper end of currently available pharmacological weight-loss treatments.

The durability of treatment is clinically important. Obesity is biologically defended, and weight regain after stopping therapy is common. In the STEP 1 extension, withdrawal of semaglutide was followed by substantial weight regain and partial reversal of cardiometabolic improvements [7]. SURMOUNT-4 showed a similar principle with tirzepatide: continued treatment maintained and extended weight loss, whereas switching to placebo led to a regain [8].

These findings should shape patient counseling. Incretin-based therapy should not be described as a brief course that permanently resets body weight. For many patients, especially those with obesity-related complications, treatment may need to be long-term. This has implications for cost, supply, monitoring, prescribing responsibility, and patient expectations. It also reinforces the importance of combining pharmacotherapy with nutrition support, resistance exercise, behavioral strategies, and management of comorbid disease.

Cardiovascular disease

The cardiovascular value of GLP-1 RAs was first established in patients with type 2 diabetes. In LEADER, liraglutide reduced major adverse cardiovascular events in patients with type 2 diabetes and high cardiovascular risk [9]. In SUSTAIN-6, semaglutide reduced cardiovascular events in a high-risk type 2 diabetes population [10]. A subsequent systematic review and meta-analysis of randomized trials supported reductions in cardiovascular mortality and kidney outcomes with GLP-1 RAs in type 2 diabetes [11].

The SELECT trial changed the discussion because it studied semaglutide 2.4 mg in patients with established cardiovascular disease and overweight or obesity, but without diabetes. Semaglutide reduced the risk of cardiovascular death, nonfatal myocardial infarction, or nonfatal stroke compared with placebo [12]. This was a major development because it showed that semaglutide could reduce cardiovascular events in a population selected by obesity and cardiovascular disease rather than hyperglycemia. It is also particularly important for the theme of this review because SELECT enrolled patients without diabetes, supporting the idea that cardiovascular benefit is not simply a consequence of improved glycaemic control [12].

The mechanisms behind cardiovascular benefit are probably mixed. Weight loss can reduce blood pressure, improve insulin resistance, lower inflammatory burden, and improve lipid-related risk [2,11,12]. GLP-1 RAs may also have effects on vascular inflammation, endothelial function, albuminuria, and atherosclerotic biology [2,11]. In practice, it may not be necessary to separate these mechanisms completely. What matters clinically is that selected patients with cardiovascular disease and excess adiposity may gain benefits beyond weight reduction alone [11,12].

This does not mean that incretin therapy replaces established cardiovascular prevention. Statins, blood pressure control, smoking cessation, antiplatelet therapy where indicated, diabetes management, and lifestyle intervention remain central. The emerging role of semaglutide is as an additional therapy for patients whose cardiovascular risk is closely linked to excess adiposity.

Heart failure with preserved ejection fraction

HFpEF is a heterogeneous syndrome. In some patients, obesity is not simply a comorbidity but a major driver of symptoms and pathophysiology. Obesity-related HFpEF is associated with increased plasma volume, systemic inflammation, insulin resistance, visceral and epicardial adiposity, impaired exercise reserve, and high symptom burden. This phenotype provides a strong rationale for targeting adiposity directly.

In STEP-HFpEF, semaglutide 2.4 mg improved heart failure-related symptoms, physical limitations, exercise function, inflammation, and body weight compared with placebo in patients with HFpEF and obesity [13]. At 52 weeks, the mean change in the Kansas City Cardiomyopathy Questionnaire clinical summary score was 16.6 points with semaglutide and 8.7 points with placebo [24]. Mean body weight changed by -13.3% and -2.6%, respectively. Semaglutide also improved six-minute walk distance and reduced C-reactive protein to a greater extent than placebo [13]. STEP-HFpEF DM extended this evidence to patients with obesity-related HFpEF and type 2 diabetes, again showing improvement in symptoms, physical limitations, exercise function, and weight [14]. This is important because patients with type 2 diabetes often lose less weight with anti-obesity pharmacotherapy than patients without diabetes and have a more complex HFpEF phenotype.

Tirzepatide has also shown benefit in HFpEF. In SUMMIT, patients with HFpEF and obesity who received tirzepatide had a lower risk of the composite of cardiovascular death or worsening heart failure than those receiving placebo, alongside improved health status [15]. The trial also showed improvement in the Kansas City Cardiomyopathy Questionnaire clinical summary score, with a mean change of 19.5 points with tirzepatide and 12.7 points with placebo at 52 weeks [15]. Secondary outcomes also supported improvements in body weight, six-minute walk distance, and inflammatory markers [15]. This suggests that incretin-based therapy may influence symptoms, functional status, inflammation, adiposity, and clinical events in obesity-related HFpEF.

These findings are still likely to be partly weight-mediated, but improvements in symptoms, exercise function, inflammatory markers, and heart failure events suggest that incretin-based therapy may also modify the inflammatory, hemodynamic, and functional phenotype of obesity-related HFpEF rather than acting only through weight reduction [13-15].

These findings reframe obesity as a therapeutic target within HFpEF management rather than as a background diagnosis. However, treatment should remain integrated with established HFpEF care. Diuretics, sodium-glucose cotransporter 2 inhibitors, blood pressure control, atrial fibrillation management, exercise rehabilitation, and treatment of sleep apnoea remain important. Incretin-based therapy adds a disease-targeted option for selected patients, but it does not remove the need for comprehensive heart failure management.

Chronic kidney disease

Chronic kidney disease in type 2 diabetes is associated with high rates of cardiovascular events, kidney failure, and mortality. GLP-1 RAs had previously shown favorable signals for albuminuria and secondary kidney outcomes, but dedicated kidney outcome evidence was needed.

The FLOW trial provided this evidence. In patients with type 2 diabetes and chronic kidney disease, once-weekly semaglutide 1.0 mg reduced clinically important kidney outcomes and death from cardiovascular causes compared with placebo [16]. This strengthens the position of semaglutide as a cardiorenal risk-reducing therapy in patients with type 2 diabetes and chronic kidney disease.

Compared with semaglutide, the kidney outcome evidence for tirzepatide is currently less mature. In an exploratory post hoc analysis of SURPASS-4, tirzepatide was associated with a slower estimated glomerular filtration rate decline and a lower occurrence of a composite kidney endpoint compared with insulin glargine in people with type 2 diabetes and increased cardiovascular risk [25]. However, this was not a dedicated chronic kidney disease outcome trial comparable to FLOW. Therefore, tirzepatide data are supportive but should be interpreted as less definitive than the semaglutide kidney outcome evidence.

The mechanisms may include improved glycaemic control, weight loss, blood pressure reduction, lower albuminuria, anti-inflammatory effects, and possible direct renal actions. It is unlikely that a single mechanism explains all observed benefits. Clinically, incretin-based therapy is most relevant for patients with overlapping metabolic, renal, and cardiovascular risk, although the strongest dedicated kidney outcome evidence currently relates to semaglutide.

This is relevant to the broader argument of the review because kidney benefit may reflect a combination of metabolic improvement, blood pressure reduction, albuminuria reduction, anti-inflammatory effects, and possible direct renal pathways, rather than weight loss alone [11,16,25].

A practical question is how GLP-1 RAs should be combined or sequenced with other kidney-protective therapies. This is particularly relevant because many patients with chronic kidney disease and type 2 diabetes are already treated with multiple cardiometabolic agents. Future studies should clarify the effects of incretin-based therapy in non-diabetic chronic kidney disease, more advanced kidney disease, and real-world populations with multimorbidity.

Metabolic dysfunction-associated steatohepatitis

Metabolic dysfunction-associated steatotic liver disease is closely linked with obesity, insulin resistance, dyslipidemia, and type 2 diabetes. The updated European Association for the Study of the Liver, European Association for the Study of Diabetes, and European Association for the Study of Obesity guidelines frame MASLD as a cardiometabolic liver disease and emphasize case-finding for fibrosis in people with cardiometabolic risk factors, particularly type 2 diabetes and obesity [17]. MASH represents the inflammatory and progressive form of this disease spectrum and can lead to fibrosis, cirrhosis, liver failure, hepatocellular carcinoma, and liver-related death. Because weight reduction can improve hepatic steatosis and steatohepatitis, incretin-based therapies are biologically plausible treatments.

Tirzepatide has shown histological benefit in MASH. In SYNERGY-NASH, patients with MASH and moderate or severe fibrosis received tirzepatide or placebo for 52 weeks. Tirzepatide was more effective than placebo for achieving resolution of MASH without worsening of fibrosis [18]. This suggests that dual incretin therapy may reduce hepatic inflammation and metabolic injury, probably through a combination of weight loss, improved insulin sensitivity, and reduced hepatic fat.

Semaglutide has also advanced in MASH. In the phase 3 ESSENCE trial, semaglutide 2.4 mg improved histological endpoints, including resolution of steatohepatitis without worsening of fibrosis and reduction in liver fibrosis without worsening of steatohepatitis [19]. Subsequent regulatory developments have further emphasized the clinical relevance of these data [20]. These histological findings strengthen the argument that incretin-based therapies may influence liver disease activity. However, the relative contributions of weight loss, improved insulin resistance, reduced hepatic fat, and direct anti-inflammatory effects remain uncertain [18,19].

This is a rapidly changing area, but caution is still required. Histological endpoints are important, but long-term clinical outcomes such as hepatic decompensation, transplantation, hepatocellular carcinoma, and liver-related mortality need longer follow-up. MASH management also requires broader metabolic risk reduction, including diabetes control, lipid management, alcohol assessment, weight management, fibrosis monitoring, and cardiovascular prevention. Incretin-based therapy may become central to MASH care, but it should not be separated from these wider priorities.

Obstructive sleep apnoea

Obstructive sleep apnoea is common in adults with obesity and contributes to daytime sleepiness, impaired quality of life, hypertension, atrial fibrillation, metabolic dysfunction, and cardiovascular risk. Weight management is an evidence-based component of adult obstructive sleep apnoea care in patients with overweight or obesity, although it is usually delivered alongside established treatments rather than as a standalone replacement [21]. Standard treatment includes weight reduction, continuous positive airway pressure, mandibular advancement devices, positional therapy, and selected surgical approaches. Until recently, there were limited pharmacological options targeting obesity-related obstructive sleep apnoea.

SURMOUNT-OSA evaluated tirzepatide in adults with moderate-to-severe obstructive sleep apnoea and obesity. Tirzepatide reduced the apnoea-hypopnoea index, body weight, hypoxic burden, high-sensitivity C-reactive protein concentration, and systolic blood pressure, while improving sleep-related patient-reported outcomes [22]. This evidence contributed to the recognition of tirzepatide as a pharmacological option for moderate-to-severe obstructive sleep apnoea in adults with obesity [23].

The obstructive sleep apnoea evidence is strongest for tirzepatide because SURMOUNT-OSA directly studied adults with moderate-to-severe obstructive sleep apnoea and obesity [22]. Other incretin-based therapies may still be relevant through weight reduction. For example, liraglutide 3.0 mg reduced apnoea-hypopnoea index compared with placebo in the SCALE Sleep Apnea trial [26]. However, semaglutide does not yet have comparable phase 3 obstructive sleep apnoea outcome evidence, so its role in this area remains mainly inferred from weight loss and broader cardiometabolic benefit rather than direct sleep apnoea trial data.

This is clinically important because tirzepatide targets one of the upstream drivers of obstructive sleep apnoea: excess adiposity. Weight loss can reduce upper-airway collapsibility, improve lung volumes, decrease rostral fluid shift, and reduce inflammatory and cardiometabolic stress. However, tirzepatide should not be seen as a universal replacement for continuous positive airway pressure. Many patients will continue to need device-based therapy, particularly if they have residual moderate-to-severe disease, severe nocturnal hypoxemia, or high cardiovascular risk. The more realistic role is combined care: treating obesity as a disease driver while continuing to monitor and manage sleep-disordered breathing.

In obstructive sleep apnoea, the mechanism is probably more directly weight-mediated than in some other disease areas, but reductions in hypoxic burden, blood pressure, and inflammatory markers suggest wider cardiometabolic relevance beyond symptom improvement alone [22].

Safety and tolerability

The most common adverse effects of incretin-based therapies are gastrointestinal. Nausea, vomiting, diarrhea, constipation, abdominal discomfort, and reduced appetite occur most often during dose escalation. These symptoms are usually mild to moderate, but they can affect adherence and may lead to discontinuation. Gradual dose escalation, dietary adjustment, hydration advice, and early review can improve tolerability.

Gallbladder and biliary disease require specific counseling. A systematic review and meta-analysis of randomized trials found that GLP-1 RA use was associated with an increased risk of gallbladder and biliary diseases, particularly at higher doses, with longer treatment duration, and when used for weight loss [27]. This may reflect rapid weight loss, altered gallbladder motility, or both. Patients should be advised to seek assessment for persistent right upper quadrant pain, fever, jaundice, or symptoms suggestive of cholecystitis.

Pancreatitis has been a long-standing concern with incretin therapies. Although causality remains debated, severe persistent abdominal pain, especially if radiating to the back or associated with vomiting, should prompt urgent assessment.

Peri-procedural management also needs consideration because GLP-1 RAs may delay gastric emptying and raise concerns about residual gastric contents and aspiration risk during anesthesia or sedation. Multisociety guidance emphasizes individualized risk assessment, particularly for patients in dose escalation, those with active gastrointestinal symptoms, and those with other causes of delayed gastric emptying [28].

Pregnancy and reproductive planning require specific counseling. Prescribing information for semaglutide advises discontinuation before planned pregnancy, while prescribing information for tirzepatide advises discontinuation when pregnancy is recognized and notes that weight loss is not recommended during pregnancy [29,30]. In practice, patients of reproductive potential should be advised to discuss pregnancy plans before starting therapy.

Lean mass loss is another important issue. Substantial weight loss reduces fat mass but can also reduce lean mass. This matters in older adults, frail patients, and patients with sarcopenic obesity. Although not specific to incretin-based therapy, evidence from older adults with obesity shows that combining weight loss with structured exercise, particularly combined aerobic and resistance exercise, can improve physical function and help preserve lean mass [31]. Prescribing should therefore include advice on resistance exercise, adequate protein intake, and maintenance of function. Weight loss that improves cardiometabolic markers but worsens strength or frailty would be a poor clinical trade-off.

In patients with diabetes, insulin or sulfonylurea doses may require adjustment to avoid hypoglycemia as glycaemic control improves. Diabetic retinopathy also deserves attention. Rapid improvement in glycemia has previously been associated with early worsening of retinopathy in susceptible patients, particularly those with poor baseline glycaemic control and pre-existing retinopathy [10]. Patients with advanced diabetic eye disease may require appropriate ophthalmic monitoring when potent glucose-lowering therapy is started.

Clinical implementation and equity

The rapid expansion of incretin-based therapy has created practical challenges. Access is strongly influenced by cost, local prescribing criteria, insurance coverage, specialist pathways, and drug availability. These barriers matter because obesity-related cardiometabolic disease often clusters with broader social and health-system disadvantage.

Patient selection should be based on disease burden, not body mass index alone. Patients with established cardiovascular disease and overweight or obesity, obesity-related HFpEF, type 2 diabetes with chronic kidney disease, MASH with fibrosis, and obstructive sleep apnoea with obesity may benefit beyond weight reduction. However, prescribing should also consider frailty, gastrointestinal tolerability, eating-disorder risk, pregnancy plans, comorbidities, patient preference, and ability to attend follow-up.

Monitoring should include weight, blood pressure, glycaemic control, renal function, gastrointestinal adverse effects, nutritional intake, physical function, and organ-specific outcomes where relevant. This aligns with obesity pharmacotherapy guidance, which emphasizes comorbidities, adverse-effect profile, reproductive considerations, cost, access, and the chronicity of obesity when selecting treatment [32]. In heart failure, volume status and diuretic requirements may change as weight and dietary intake change. In obstructive sleep apnoea, repeat sleep assessment may be needed to determine residual disease. In MASH, fibrosis monitoring remains central because histological improvement does not remove long-term liver risk [17]. In patients with chronic kidney disease, ongoing assessment of kidney function and albuminuria should be guided by chronic kidney disease risk stratification and disease-specific monitoring principles [33].

A better way to frame treatment is that pharmacotherapy can make lifestyle change more achievable by reducing appetite, cravings, and the biological drive toward weight regain. The best outcomes are likely to come from combining medication with nutrition support, resistance exercise, behavioral strategies, sleep optimization, and management of cardiovascular risk factors. Practical prescribing considerations for incretin-based therapy in obesity-related multimorbidity are summarized in Table 2.

**Table 2:** Practical considerations when prescribing incretin-based therapy in obesity-related multimorbidity.

Issue	Practical implication	Supporting evidence or rationale
Gastrointestinal symptoms	Nausea, vomiting, diarrhea, constipation, and abdominal discomfort are common, particularly during dose escalation. Slower titration, dietary adjustment, and early review may improve tolerability.	Reported consistently across obesity and diabetes trials, including STEP 1 and SURMOUNT-1 [5,6]
Gallbladder and biliary disease	Patients should be counseled about biliary colic, cholecystitis symptoms, and the possibility of higher risk during rapid weight loss.	Supported by randomized-trial meta-analysis showing increased gallbladder and biliary disease risk with GLP-1 RAs [27]
Weight regain after discontinuation	Patients should be told that treatment discontinuation is commonly followed by weight regain and partial reversal of cardiometabolic improvements.	Demonstrated after semaglutide withdrawal, tirzepatide withdrawal and continuation studies [7,8]
Diabetes medication adjustment	Insulin and sulfonylurea doses may need review to reduce hypoglycemia risk as glycemic control and dietary intake change.	Mechanistically expected from glucose-lowering effects and relevant to diabetes prescribing [2-4]
Diabetic retinopathy	Patients with advanced diabetic retinopathy or rapid glycemic improvement may need closer ophthalmic monitoring.	Relevant to potent glucose-lowering, particularly in patients with poor baseline glycaemic control [10]
Peri-procedural care	Delayed gastric emptying may matter before anesthesia or sedation, particularly during dose escalation or in patients with gastrointestinal symptoms.	Related to GLP-1 RA gastrointestinal pharmacology and multisociety perioperative guidance [2,3,28]
Pregnancy and reproductive planning	Patients of reproductive potential should discuss pregnancy plans before starting therapy, and local product information should be followed.	Supported by semaglutide and tirzepatide prescribing information [29,30]
Lean mass and function	Weight loss should be accompanied by resistance exercise, adequate protein intake, and functional monitoring, especially in older or frail patients.	Clinically important because substantial weight loss can include loss of lean mass [31]
Access and equity	Cost, supply, and local prescribing restrictions may widen disparities if access is limited to patients with greater resources.	Important because obesity-related cardiometabolic disease is socially patterned

Future directions

The incretin field continues to evolve. Retatrutide, a triple agonist targeting GIP, GLP-1, and glucagon receptors, has shown substantial weight reduction in phase 2 obesity research [34]. Oral incretin therapies and new combination approaches may also change future practice if efficacy, safety, and adherence are confirmed.

Several research questions remain. First, the relative contribution of weight loss and direct incretin signaling to organ-specific benefit is not fully defined. Second, optimal treatment duration is uncertain, although withdrawal studies strongly suggest that ongoing therapy is usually required to maintain benefit. Third, long-term safety data are needed in older adults, frail patients, patients with advanced organ disease, and patients with multiple comorbidities. Fourth, comparative effectiveness against bariatric surgery, sodium-glucose cotransporter 2 inhibitors, finerenone, continuous positive airway pressure, and emerging MASH therapies remains unclear.

Future trials should prioritize hard clinical outcomes. These include cardiovascular death, heart failure hospitalization, kidney failure, liver decompensation, transplantation, hepatocellular carcinoma, and all-cause mortality. Trials should also include more diverse populations, including patients from socioeconomically disadvantaged backgrounds. Without broader representation, the patients most affected by obesity-related multimorbidity may remain underrepresented in the evidence base.

Limitations 

This review has limitations. It is a narrative review and does not use systematic review methodology. Although priority was given to high-impact randomized trials, cardiovascular outcome trials, systematic reviews, clinical practice guidelines, regulatory sources, and prescribing information, the literature was not screened using a formal Preferred Reporting Items for Systematic reviews and Meta-Analyses (PRISMA) process. No formal risk-of-bias assessment or quantitative synthesis was performed. The conclusions should therefore be interpreted as a clinically oriented synthesis rather than a definitive systematic assessment of all available evidence.

The evidence base is also evolving quickly. New trial results, longer-term follow-up, real-world safety data, and regulatory decisions may alter the clinical positioning of these therapies. Some indications, particularly in MASH, are partly supported by histological surrogate endpoints, and longer-term clinical outcomes remain necessary. Finally, trial populations may not fully represent older adults, frail patients, patients with advanced multimorbidity, or those facing socioeconomic barriers to accessing care.

## Conclusions

Incretin-based therapies have moved beyond their original role as glucose-lowering and weight-loss drugs and are increasingly relevant to the treatment of obesity-related organ disease. Semaglutide and tirzepatide now have evidence across obesity, atherosclerotic cardiovascular disease, obesity-related HFpEF, chronic kidney disease, MASH, and obstructive sleep apnoea. However, the strength of evidence varies by condition, and the relative contribution of weight-dependent and weight-independent mechanisms remains incompletely defined. These therapies should therefore be integrated into chronic disease pathways for selected patients, with careful attention to patient selection, tolerability, long-term treatment requirements, safety monitoring, cost, access, and multidisciplinary care.
